# Genome wide characterization of simple sequence repeats in watermelon genome and their application in comparative mapping and genetic diversity analysis

**DOI:** 10.1186/s12864-016-2870-4

**Published:** 2016-08-05

**Authors:** Huayu Zhu, Pengyao Song, Dal-Hoe Koo, Luqin Guo, Yanman Li, Shouru Sun, Yiqun Weng, Luming Yang

**Affiliations:** 1 College of Horticulture, Henan Agricultural University, 63 Nongye Road, Zhengzhou, 450002 China; 2 Horticulture Department, University of Wisconsin, Madison, WI 53706 USA; 3 US Department of Agriculture/Agricultural Research Service, Vegetable Crops Research Unit, 1575 Linden Drive, Madison, WI 53706 USA

**Keywords:** SSR, Watermelon, Comparative genomics, Synteny, Cucurbits, Genetic diversity

## Abstract

**Background:**

Microsatellite markers are one of the most informative and versatile DNA-based markers used in plant genetic research, but their development has traditionally been difficult and costly. The whole genome sequencing with next-generation sequencing (NGS) technologies provides large amounts of sequence data to develop numerous microsatellite markers at whole genome scale. SSR markers have great advantage in cross-species comparisons and allow investigation of karyotype and genome evolution through highly efficient computation approaches such as in silico PCR. Here we described genome wide development and characterization of SSR markers in the watermelon (*Citrullus lanatus*) genome, which were then use in comparative analysis with two other important crop species in the Cucurbitaceae family: cucumber (*Cucumis sativus* L.) and melon (*Cucumis melo* L.). We further applied these markers in evaluating the genetic diversity and population structure in watermelon germplasm collections.

**Results:**

A total of 39,523 microsatellite loci were identified from the watermelon draft genome with an overall density of 111 SSRs/Mbp, and 32,869 SSR primers were designed with suitable flanking sequences. The dinucleotide SSRs were the most common type representing 34.09 % of the total SSR loci and the AT-rich motifs were the most abundant in all nucleotide repeat types. In silico PCR analysis identified 832 and 925 SSR markers with each having a single amplicon in the cucumber and melon draft genome, respectively. Comparative analysis with these cross-species SSR markers revealed complicated mosaic patterns of syntenic blocks among the genomes of three species. In addition, genetic diversity analysis of 134 watermelon accessions with 32 highly informative SSR loci placed these lines into two groups with all accessions of *C.lanatus* var*. citorides* and three accessions of *C. colocynthis* clustered in one group and all accessions of *C. lanatus* var*. lanatus* and the remaining accessions of *C. colocynthis* clustered in another group. Furthermore, structure analysis was consistent with the dendrogram indicating the 134 watermelon accessions were classified into two populations.

**Conclusion:**

The large number of genome wide SSR markers developed herein from the watermelon genome provides a valuable resource for genetic map construction, QTL exploration, map-based gene cloning and marker-assisted selection in watermelon which has a very narrow genetic base and extremely low polymorphism among cultivated lines. Furthermore, the cross-species transferable SSR markers identified herein should also have practical uses in many applications in species of Cucurbitaceae family whose whole genome sequences are not yet available.

**Electronic supplementary material:**

The online version of this article (doi:10.1186/s12864-016-2870-4) contains supplementary material, which is available to authorized users.

## Background

Watermelon (*Citrullus lanatus*) is an important horticultural crop and one of the most consumed fresh fruits globally. It belongs to the genus *Citrullus*, which contains four diploid species: *Citrullus lanatus* (Thunb.) Mat-sum. & Nakai*, C. colocynthis* (L.) Schrad, *C. ecir rhosus* Cogn. and *C. rehmii* De Winter [1, 2]. Among these four species, *Citrullus lanatus* includes the cultivated watermelon (*C. lanatus* var*. lanatus*) which thrives in West Africa and has been cultivated widely worldwide (also called ‘egusi’ melon) and the preserving melon (*C. lanatus* var. *citroides*) that is grown in Southern Africa (also called ‘tsamma’ melon) [3, 4], and *C. colocynthi* (‘bitter apple’) is a perennial species grown in sandy areas throughout northern Africa, south-western Asia, and the Mediterranean [2, 5]. The long term domestication and selection for desirable horticultural qualities has made the cultivated watermelon with a narrow genetic base and susceptibility to a large number of diseases and pests [6]. Evaluating the phylogenetic relationships among different species in *Citrullus* genus will help us for improving watermelon cultivars in diseases resistance [1]. Watermelon has a small genome of 425 Mb, and the genome of the elite Chinese watermelon line 97103 [7] and the American heirloom watermelon cultivar Charleston Gray have been sequenced and released in cucurbit genomics database (www.icugi.org). The availability of these genomic resources of watermelon have greatly promoted the fundamental researches including the development of molecular markers and genetic map construction [8, 9], gene/QTL mapping [10, 11], molecular breeding, and comparative genomics [12].

Microsatellites or simple sequence repeats (SSRs), are one of the most commonly used marker in many genetic applications since the early 1990s including mapping, fingerprinting, genetic diversity and population structure analysis [13–16]. Because of their reproducibility, multi-allelism, co-dominance, relative abundance, good genome coverage and versatile platforms to genotype, the use of microsatellites is likely to continue to be used for some years to come. Furthermore, they are comparatively cheap to genotype and provide more population genetic information per marker than bi-allelic markers such as single nucleotide polymorphisms (SNP) [17, 18]. A single set of microsatellite markers can be used to genotype several related species, but SNP markers in general lack cross-species utility, and are therefore only suitable for population and paternity studies in a single species [19–21]. The microsatellite loci can be detected both in genomic sequences and expressed sequence tag (EST), which were named genomic SSRs and EST-SSR. EST-SSRs are useful for genetic analysis, but their relatively low polymorphism and the high possibility of no gene-rich regions in the genome are limitations to their use. In contrast, genomic SSRs are highly polymorphic and tend to be widely distributed throughout the genome, resulting in better map coverage [22].

With the rapid development of sequencing technologies, whole-genome sequences (WGS) are becoming increasingly available. These DNA sequences are valuable resources for SSR development and genome wide identification of SSR have been investigated in many plant species, such as cucumber [23], foxtail millet [24] and *Brassica* [25]. Together with the advantage of in silico analysis, this approach has the potential to develop highly polymorphic SSR markers to suit various applications such as comparative studies in species where limited or no sequence information is available [12, 26, 27]. However, large scale development of microsatellite markers was not realized until the whole genome sequence of watermelon was available [7]. Recently Ren et al. [8] identified 13,744 putative SSR loci and 1877 unique SSRs with long repeat motifs were selected for polymorphism analysis and genetic map construction. The usefulness of these watermelon microsatellite markers has already been demonstrated in recent linkage mapping [11] and genetic diversity studies [28]. Despite such progresses, the number of robust, informative and user-friendly markers publicly available for watermelon is still insufficient for some applications, particularly considering the low intra-specific polymorphism level of microsatellite markers in watermelon. The availability of microsatellite markers distributed throughout the genome would facilitate the development of high resolution maps or rapid saturation of target genomic regions, which is instrumental for applications like positional gene cloning and detailed comparative mapping. Such molecular resources would benefit the watermelon research and breeding community.

Watermelon (2n = 2x = 22) belongs to the Cucurbitaceae family which includes several economically important species, such as melon (*Cucumis melo*, 2n = 2x = 24) and cucumber (*Cucumis sativus* L., 2n = 2x = 14). Draft genome assemblies for the three species are now publicly available [23, 27, 29, 30] and the availability of large numbers of molecular markers has made it possible to define more clearly syntenic relationships among them. To access the nature of evolutionary events leading to modern cucurbit genome structures, Huang et al. [29] established the syntenic relationship between cucumber and watermelon by alignment of 136 watermelon marker sequences mapped from watermelon linkage groups in 9930 cucumber draft genome and Guo et al. [7] investigated the chromosome-to-chromosome relationships within the Cucurbitaceae family by comparative mapping and identified the complicated syntenic patterns illustrated as mosaic chromosome-to-chromosome orthologous relationships among watermelon, cucumber and melon. In the genus *Cucumis* genus, syntenic relationships among chromosomes of cucumber, melon and *C. hystrix* have been extensively analysed [12, 27, 30, 31]. For example, 91 syntenic blocks were divided between cucumber and melon, and 53 syntenic blocks were identified betweeen cucumber and *C. hystrix* by comparative mapping and comparative fluorescence in situ hybridization (FISH). These findings revealed a high degree of complexity of structure rearrangements after cucumber and melon diverging from their common ancestor. However, the syntenic relationship and chromosomal rearrangements between watermelon with cucumber and melon are still largely fragmented and incomplete.

In this study, we identified genome wide SSR in watermelon and characterized the distribution and frequency of different motifs and repeats. We further identified cross-species transferable SSR markers in cucumber and melon by in silico PCR analysis, and established syntenic relationships between watermelon and cucumber, as well as between watermelon and melon chromosomes based on shared SSR markers. In addition, 32 highly informative SSR markers were identified and used to evaluate the genetic diversity and population structure of 134 *Citrullus* accessions including *C. lanatus* var. *lanthus*, *C. lanatus* var. *citroides* and *C. colocynthis*.

## Results

### The frequency and distribution of different SSR types in watermelon genome

**Fig. 1:**
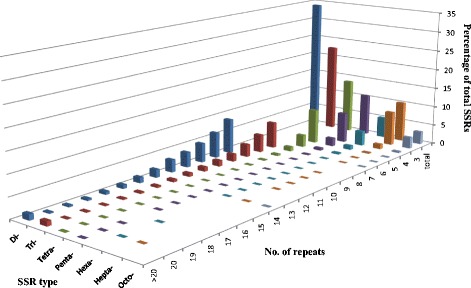
Distribution of SSR motif repeat numbers and relative frequency in watermelon genome. The vertical axis shows the abundance of microsatellites that have different motif repeat numbers (from 3 to > 20), which are discriminated by legends of different colours

A total of 39,523 microsatellite sequences were identified in the released 353.5 Mb genomic sequences of East Asia watermelon cultivar 97103 with more conserved criteria than that in cucumber [23]. The total length of all SSR sequences was estimated to be 0.28 % of the draft genome assembly with an average of 111 SSR/Mb. Among different nucleotide types, the microsatellite frequency was negatively correlated with the number of nucleotide. For example, dinucleotide repeats were the most abundant accounting for 34.09 % of the total SSR loci discovered, followed by tri- (22.64 %) and tetra- (13.83 %), and octonucleotides were the least frequent repeat types (3.27 %) (Table 1). We also investigated the SSR motif distribution with regard to repeat number. For all seven SSR types, microsatellite frequency decreased as the number of repeat units increased, which was more obvious with longer SSR motifs (Fig. 1). As a consequence, the mean repeat number in dinucleotides (12.29) was about four times the number of hepta- and octonucleotide (3.19 and 3.14 respectively) (Table 1).

**Table 1:** The distribution of different nucleotide repeats in watermelon genome

Nucleotide	Number of loci identified	Frequency (%)	Mean repeat number	Number of loci primer designed	Percentage SSRs suitable for primer design (%)
Di	13474	34.09	12.29	11353	84.26
Tri	8947	22.64	10.56	7718	86.26
Tetra	5465	13.83	5.72	4552	83.29
Penta	4205	10.64	4.43	3681	87.54
Hexa	2082	5.28	4.43	1938	93.08
Hepta	4059	10.27	3.19	3412	84.06
Octo	1291	3.27	3.14	1156	89.54
Total	39523	100		33810	85.55

**Fig. 2:**
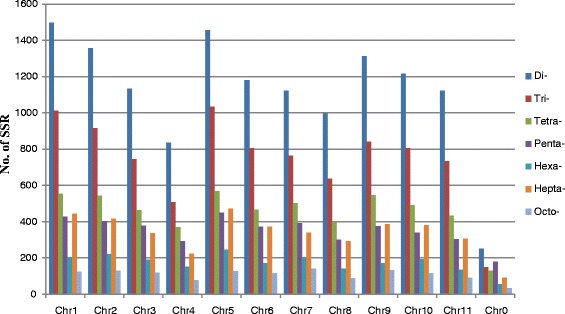
The distribution of SSR repeat types on each chromosome in watermelon. The vertical axis shows the number of microsatellites from dinucleotide to octonucleotide which are discriminated by different colours. The horizontal axis show different chromosomes of watermelon and chr0 represent all the chromosomal unanchored scaffolds

We examined the nucleotide composition of each motif type and found that some combinations of nucleotides were more prevalent than others in each class. For example, the AT motif was dramatically overrepresented in dinucleotide motifs, and it was also the most frequent motif in the entire watermelon genome, which accounting for 25.07 % of the total SSR loci discovered. Similarly, the AAT, AAAT, AAAAT, AAAAAT, AAAAAAT and AAAAAAAT were the most abundant repeats types in each class (Additional file 1: Figure S1). We further investigated the frequency and distribution of different SSR types in each watermelon chromosome. The frequency of microsatellite loci was not correlated with the chromosome size (Fig. 2). For example, chromosome 6 had the highest density of 129.03 SSR/Mb, while it was one of the smallest chromosomes of watermelon (Table 2). The largest number of microsatellite was detected on chromosome 5 (4349), followed by chromosome 1 (4264) and 2 (3980), and the least SSR number was located on chromosome 4 (2456). There were even 889 SSR loci detected on these scaffolds not yet anchored to any of the 11 chromosomes (designated as chr0) with a very low density of 36 SSR/Mb.

**Table 2:** The distribution of nucleotide repeats on different chromosomes

Nucleotide	Chr1	Chr2	Chr3	Chr4	Chr5	Chr6	Chr7	Chr8	Chr9	Chr10	Chr11
Di-	1496	1355	1133	836	1456	1180	1121	994	1313	1217	1122
Tri-	1011	915	745	508	1033	805	765	636	841	806	732
Tetra-	555	542	464	369	567	467	502	399	546	492	433
Penta-	427	402	379	291	449	373	392	299	374	338	302
Hexa-	205	221	190	151	245	172	202	141	172	192	136
Hepta-	445	415	336	224	472	373	340	291	385	381	306
Octo-	125	130	118	77	127	116	140	87	132	115	90
Total	4264	3980	3365	2456	4349	3486	3462	2847	3763	3541	3121
Chr. Size (Mb)	34.08	34.41	28.94	24.32	33.72	27.02	31.48	26.15	34.99	28.42	27.11
Density (SSRs/Mb)	125.11	115.65	116.28	101.00	128.99	129.03	109.98	108.88	107.55	124.60	115.14

The genomics DNA sequences containing these microsatellites were screened for PCR primer design using Primer3, and 33,810 SSR microsatellite loci contained suitable flanking sites for SSR primer design. Finally, we designed 32,869 SSR primers with some SSR loci included in the same primers as compound SSRs. The exact positions of these SSRs in the watermelon chromosomes, as well as information on repeat motifs and expected PCR product length are presented in Additional file 2: Table S2.

### Comparative analysis of watermelon SSR markers in cucumber and melon genome

To identify genome wide cross-species watermelon SSR markers, all 32,869 SSR markers were used for in silico PCR analysis using cucumber and melon draft genome sequences as the templates. We identified 832 (2.53 %) SSR markers with a single in silico PCR product in the seven pseudochromosomes of cucumber Gy14, whereas 59 SSR markers had at least two products and ClSSR20036 had the largest number of 25 products which were distributed on all seven cucumber chromosomes. These cross-species SSR markers covered 190.42 Mb accounting for 98.87 % of the cucumber assembly. The physical positions of common markers between watermelon and cucumber are presented in Additional file 2: Table S3. The number of cross-species SSR markers in melon genome was comparable to that in cucumber. Totally, 925 (2.81 %) of all SSR markers had one in silico PCR products in melon genome assembly, and 44 SSR markers had at least two products. The cross-species SSR markers in melon were spanned 310.94 Mb accounting for 98.29 % of melon genome assembly. The physical positions of cross-species markers between melon and watermelon are presented in Additional file 2: Table S4.

The distribution and frequency of cross-species SSR markers on each chromosome in cucumber and melon were also investigated. In cucumber, there was an average of 119 common SSR markers on each chromosome with a density of 4.32SSR/Mb. Chromosome C3 had the largest number of 183 common markers and C7 had the least number of 68 common markers, which was largely consistent with the physical lengths of the two chromosomes. In melon, of these 925 cross-species SSR markers, 63 were located in the unassembled chromosome, so the remaining 862 common SSR markers were mapped in 12 chromosomes with an average of 72 SSR markers on each chromosome (Additional file 2: Table S4). Melon chromosome VI had the largest number of 98 common markers and the highest density of 3.31SSR/Mb. Though the chromosome X had the least number of 45 common markers, the chromosome V had the lowest density of 2.0 SSR/Mb. There was no direct correlation between chromosome size and number of cross-species SSR markers. This is probably more dependent on the conservation of syntenic regions between species in particular chromosomes.

**Fig. 3:**
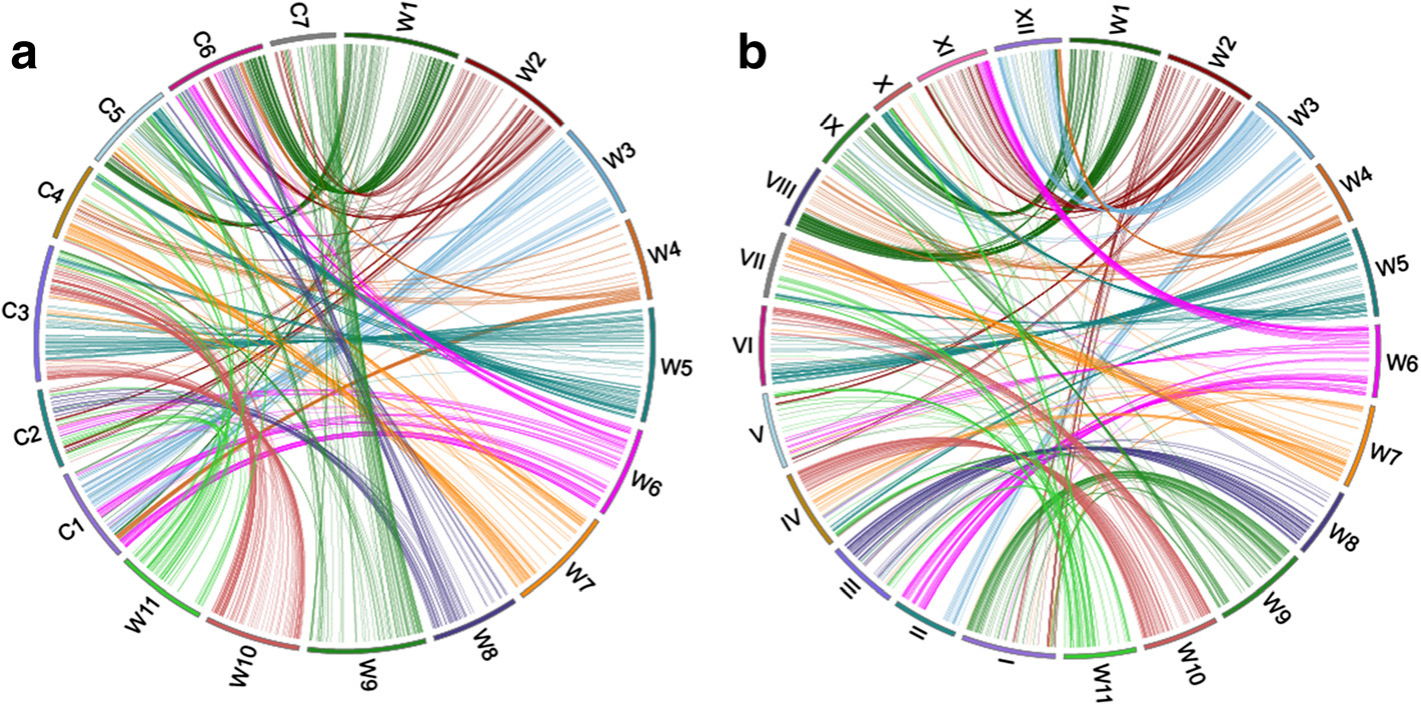
Syntenic relationships of watermelon with cucumber (**a**) and melon (**b**) chromosomes. Chromosome synteny between watermelon and cucumber is based on 821 cross-species markers (A); synteny between watermelon and melon is based on 850 cross-species markers. W1-W11 represent watermelon eleven chromosomes, C1-C7 represent cucumber seven chromosomes and I-XII represent melon twelve chromsomes. Syntenic blocks are connected by with the same colour lines from watermelon chromosomes

By comparing the two cross-species SSR marker sets in cucumber and melon, 448 SSR markers were further identified shared among all three genomes (Additional file 2: Table S5). Within each chromosome, fewer markers were found around the centromeres in watermelon; most of the common SSR markers were distally distributed on each chromosome (Fig. 3). The expected in silico PCR products of 448 SSR markers in watermelon genome were also used to BLAST search in EST, unigenesand CDS database of watermelon, respectively. Of these, 21 SSR markers were expressed in EST database, 19 SSR markers were expressed in unigene database, and 210 of them were located in the coding regions suggesting these SSR markers are related to gene function (Additional file 2: Table S5). For different nucleotides repeats contained in the 448 SSR markers, the dinucleotides had the highest frequency of 87 (34.42 %), followed by tri- 64 (27.27 %) and hepta- 20 (14.29 %). The frequency of different motifs was further compared in the same nucleotides repeat. Among the dinucleotides, AG/CT had the most abundant with a frequency of 59.12 %, followed by AT/TA (33.33 %) and AC/GT (7.55 %), while AAG/CTT was the most abundant in trinucleotides with a frequency of 50.79 %.

### Chromosome synteny of watermelon with cucumber and melon

Of these 832 cross-species SSR markers between watermelon and cucumber, 11 were located on chr0 of watemelon. Based on their chromosomal positions of the remaining 821 SSR markers in both genomes, syntenic relationships between watermelon and cucumber chromosomes could be directly inferred and visualized in Fig. 3A. The main syntenic chromosomes between watermelon and cucumber revealed complex patterns for different chromosomes, and the main syntenic relationships could be listed in Table 3. Watermelon chromosome W3 and W10 had the simplest syntenic pattern with cucumber, and each of them was mainly syntenic to two cucumber chromosomes. For example, W3 had 55 shared SSR markers with cucumber, 52 of them were located on C1 and the remaining three SSR markers (ClSSR08129, ClSSR08186 and ClSSR08224) were located on C5 (Fig. 3A and Additional file 2: Table S3). Watermelon chromosome W2 and W11 showed the most complicated patterns and each of them were syntenic to five cucumber chromosomes. Chromosome W9 was syntenic to four cucumber chromosomes and the remaining six watermelon chromosomes had similar syntenic pattern with each of them corresponding to three cucumber chromosomes. According to the cross-species SSR markers sharing continuous physical positions on both genomes, the 11 watermelon chromosomes were further divided into different syntenic blocks with each block containing at least three SSR markers. 84 syntenic blocks could be recognized, 44 of which were collinear, and the remaining 40 blocks showed inversions between watermelon and cucumber genomes (Additional file 2: Table S3). Each watermelon chromosome contained 5–11 discrete syntenic blocks. The largest block was WCB73 in chromosome W10 that spanned 7.70 Mb in cucumber chromosome C3. The watermelon syntenic block WCB6 had the largest number (55) of shared SSR markers which were collinear between watermelon W1 and cucumber C6.

**Table 3:** Major syntenic chromosomes among watermelon, cucumber and melon

Watermelon	Cucumber	Melon
W1	C1(14), C5(35), C6(60)	VIII(69), IX(31), XII(20)
W2	C2(26), C4(3), C5(4), C6(22), C7(15)	I(17), V(18), X(3), XI(42)
W3	C1(52), C5(3)	II(15), IX(4), XII(38)
W4	C1(19), C4(17), C6(10)	VIII(23), XII(15)
W5	C3(67), C4(13), C5(33)	IV(13), VI(58), VII(11), X(28)
W6	C1(45), C2(11), C6(29)	II(41), V(12), XI(35)
W7	C3(12), C4(28), C5(13)	I(5), IV(14), VII(32)
W8	C2(33), C5(4), C6(28)	III(57)
W9	C3(9), C5(11), C6(4), C7(48)	I(44), IV(6), IX(14), V(3)
W10	C3(86), C4(5)	IV(47), VI(35), VII(4)
W11	C2(11), C3(8), C4(13), C5(12), C6(6)	III(7), IV(6), V(19), VII(17), IX(5), X(11)

The number in the bracket means the shared SSR markers on these chromosomes in melon and cucumber. The syntenic chromosomes with less than three shared markerswere not listed here

Similar comparison was carried out using the cross-species SSR markers between watermelon and melon. Of the 925 cross-species SSR markers between the two genomes, 850 had unambiguous chromosome locations which were used to infer the syntenic relationships between this two species (Fig. 3B and Additional file 2: Table S4). Despite of the similar chromosome numbers in melon and watermelon, the chromosome synteny between them was rather complicated (Additional file 2: Table S4) which was consistent with their far-away phylogenetic distance. In most cases, each watermelon chromosome was syntenic to three melon chromosomes. Watermelon chromosome W11 had the most complicated syntenic pattern which was composed of blocks corresponding to seven melon chromosomes, while W8 showed the simplest pattern which was only syntenic to two melon chromosomes. The whole chromosome W8 was almost syntenic to melon III except for the topmost with two SSR markers (ClSSR21281 and ClSSR21359) that were syntenic to melon IX, but from a closer look, 2 and 3 of the 5 blocks (WMB51-55) in W8 were collinear and inverted to melon chromosome III, respectively (Additional file 2: Table S4). Among the 81 syntenic blocks assigned, 48 were collinear between the two genomes. For example, 43 shared SSR markers in block WMB70 on the end of watermelon W10 were completely collinear with melon chromosome IV except one marker ClSSR29104 which located on chr0, and this block covered 8.96 and 7.35 Mb in watermelon and melon, respectively (Additional file 2: Table S4). Furthermore, we identified 25 syntenic blocks shared in three genomes which were distributed on 10 watermelon chromosomes (Additional file 2: Table S6), indicating these genomic regions are highly conserved during chromosome evolution.

**Fig. 4:**
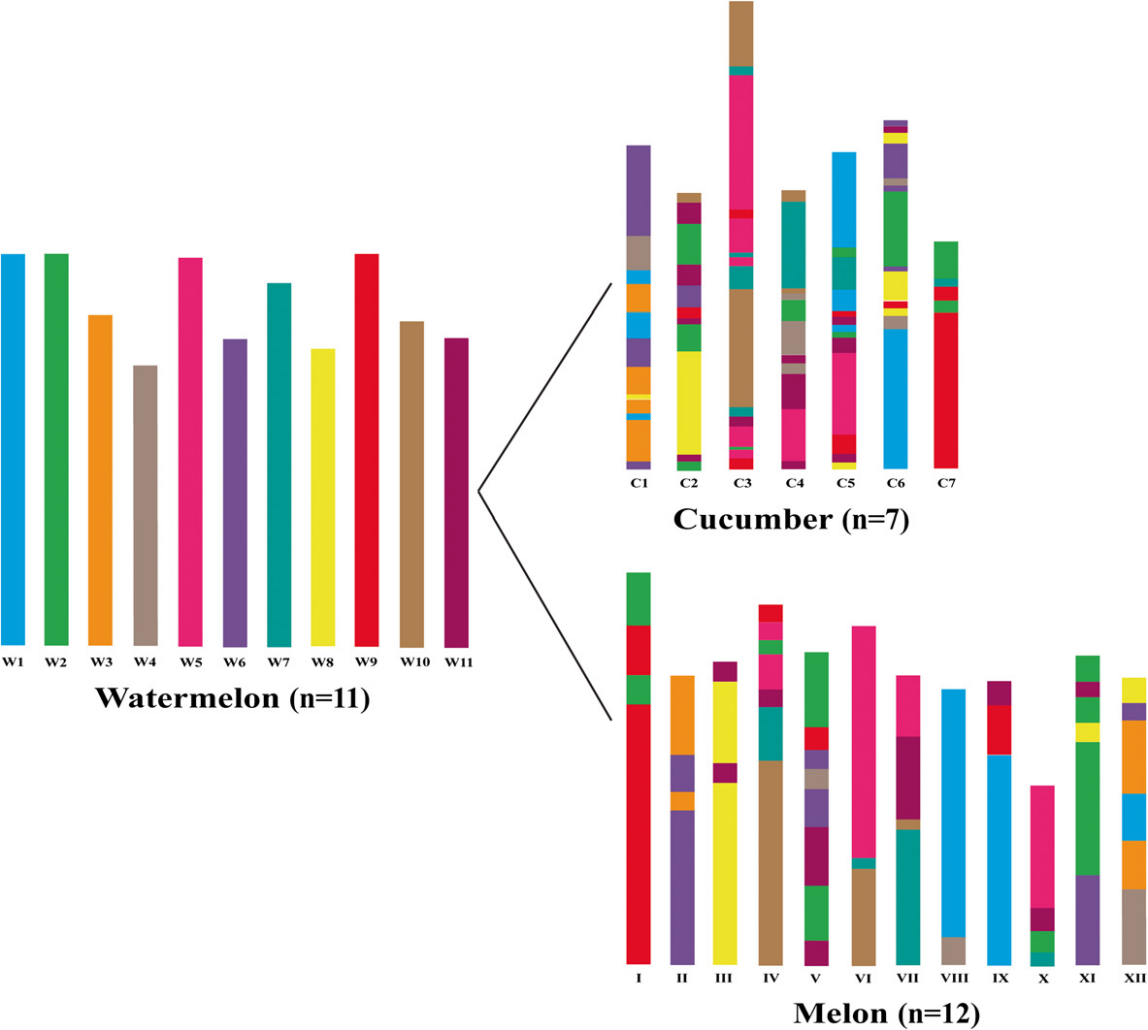
A syntenic block view of cucumber and melon chromosomes composted of watermelon chromosomes. The different colours represent the eleven chromosomes of watermelon. The mosaic colour pattern of cucumber and melon chromosomes indicated seven cucumber chromosomes and twelve melon chromosomes composed of syntenic blocks from different watermelon chromosomes

Previous studies have revealed that watermelon was diverged from the lineage leading to melon and cucumber in the Cucurbitaceae family approximately 20 million years ago [32, 33]. To better understand chromosome evolution in cucurbit species, the watermelon based syntenic block view of cucumber and melon chromosomes were developed in Fig. 4. The arrangement of watermelon syntenic blocks across seven cucumber chromosomes and twelve melon chromosomes indicated complicated mosaic patterns of chromosome evolution in species of the Cucurbitaceae family. In the melon genome, chromosomes II, III, VIII and XI were syntenic to two watermelon chromosomes, while the remaining eight chromosomes contained syntenic blocks corresponding to more than three watermelon chromosomes (Fig. 4). Compared with melon, the syntenic blocks in cucumber were even more complicated with each cucumber chromosome containing syntenic blocks from more than two watermelon chromosomes (Fig. 4). For example, cucumber C7 was largely syntenic to watermelon W2 and W9, while C6 was composed of segment regions from seven watermelon chromosomes.

**Fig. 5:**
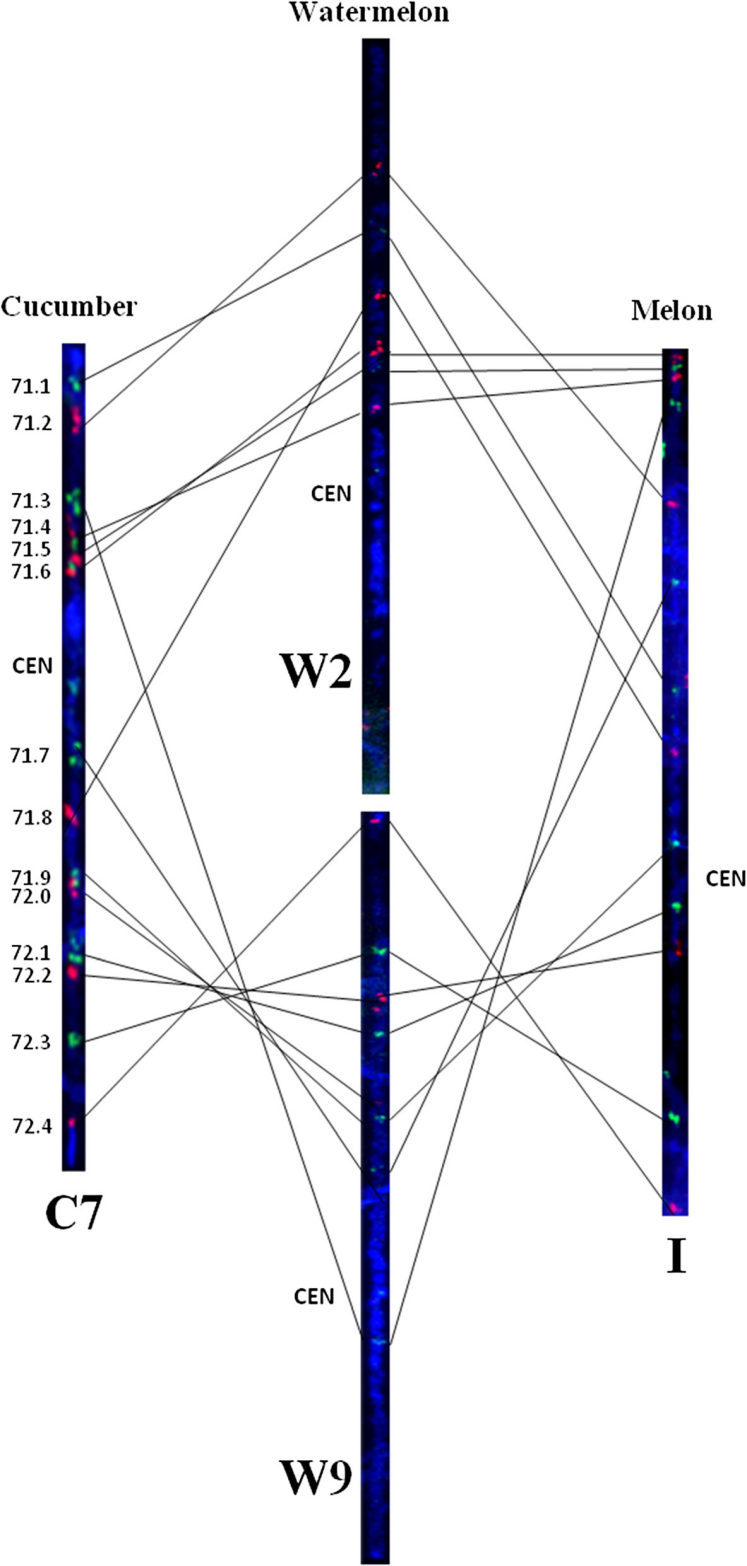
Comparative pachytene FISH analysis of cucumber (C7), melon (I) and watermelon (W2 and W9). Fourteen fosmid probes identified on cucumber chromosome C7 and melon chromosome I in a previous study (Yang et al, [12]) were used to detect their location in watermelon for verifying the results by comparative mapping. CEN indicates the putative centromere location

We verified the SSR-based syntenic relationships among the three species using fluorescence *in situ* hybridization (FISH) and cucumber C7 as an example. 14 cucumber fosmid probes located on C7 were selected in this study (Additional file 2: Table S7), where these fosmid probes have been used to confirm the synteny in different *Cucumis* species in our previous studies [12, 27]. All 14 fosmid probes detected single hybridization signal in watermelon, while they were located on two different chromosomes W2 and W9. Five probes (71.1–71.2, 71.4–71.6) from short arm and one fosmid (71.8) from long arm of cucumber C7 were located on watermelon chromosome W2, and the remaining eight probes were detected on chromosome W9 (Fig. 5). Two inversions were detected between watermelon W2 and cucumber C7 which were in accordance with block WCB8 and WCB9, indicating this was completely consistent with the results from in silico comparative mapping. The large inversion block WCB62 between the top of watermelon W9 and the end of cucumber C7 spanned almost 8 Mb in watermelon, which was also confirmed by FISH mapping using probes 71.9–72.4. Compared with cucumber, six probes on watermelon W2 were collinear with melon I, which were divided into two genomic regions in accordance with blocks WMB8 and WMB9. Three inversions were detected between watermelon W9 and melon I by the remaining fosmid probes, which were associated with the blocks WMB56, WMB 58 and WMB60. Thus, the results of FISH mapping were completely consistent with these finding revealed by comparative mapping using cross-species SSR markers.

### Application of SSR marker in watermelon genetic diversity and population structure analysis

One hundred ninety two SSR markers were selected from eleven watermelon chromosomes to test their polymorphism in six genotypes of cultivated watermelon from diverse geographical origins (Additional file 2: Table S8). Among them, 91 (47.40 %) were polymorphic among the six accessions, while almost half of them (47.25 %) had dinucleotide repeats, followed by tri- (17.58 %) and tetra- (8.79 %). To identify and validate an appropriate set of SSR markers for characterizing *C. lanatus* germplasm collections, 32 highly informative SSR markers were selected with at least two SSR primers on each chromosome (Additional file 1: Figure S2). These markers were used for fingerprinting in a germplasm panel of 134 accessions (Additional file 2: Table S1) of *Citrullus* genus including *C. colocynthis*, *C. lanatus* var. *lanatus*, *C. lanatus* var. *citorides.* The 32 SSR markers detected a total of 151 alleles with an average of 4.72 alleles per marker. The number of observed alleles (Na) ranged from two to eight, the observed heterozygosity (Ho) from 0.03 to 0.47, and Shannon’s information index (I) from 0.52 to 1.41. The PIC (polymorphic information content) value for each locus ranged from 0.22 to 0.64 with an average of 0.46 (Additional file 2: Table S9).

**Fig. 6:**
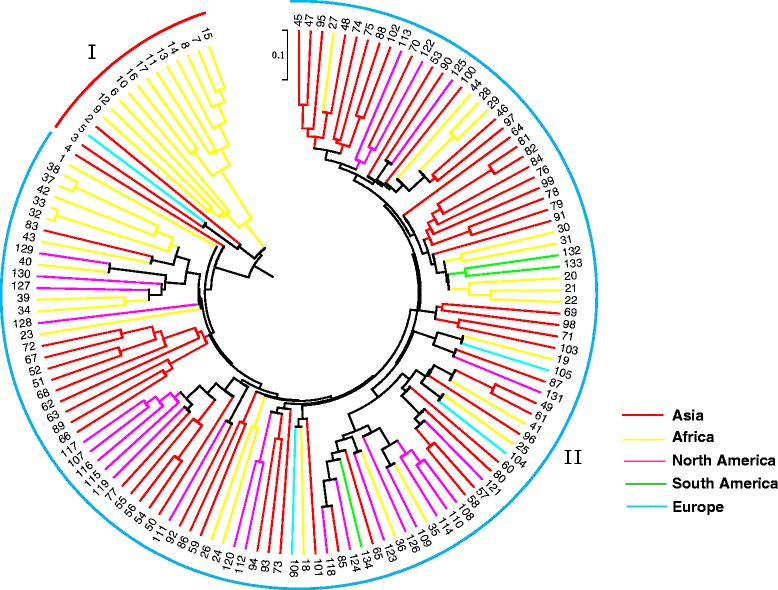
The UPGMA phylogenetic tree of the 134 accessions. The phylogenetic analysis showed 134 accessions were classified into two groups: group I and II. The colour branches represent the accessions collected from different continents. The number 1–134 represent waermelon accessions W1 to W134 in Additional file 2: Table S1. Among them, 1–5 belong to *C. colocynthis*, 6–17 belong to *C. lanatus* var. *citroides* and 18–134 belong to *C. lanatus* var. *lanatus*

**Fig. 7:**
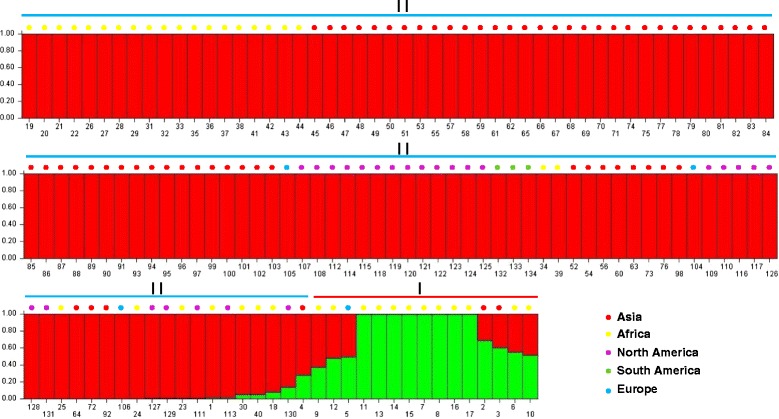
Population structure of 134 accessions in watermelon by Model-based analysis. Scale of Y axis represents the percent of genetic components, and the X axis represents the different watermelon accession. The colour dots in the top of these bar plots represent the origin of these accessions, and the latin number (I and II) corresponds to the predefined phylogenetic tree in Fig. 6

To estimate the genetic diversity among these *Citrullu*s accessions, we constructed a phylogenetic tree using the UPGMA method and two clusters were delineated. The first cluster contained all twelve *C. lanatus* var*. citorides* accessions and three *C. colocynthis* acessions (W2, W3 and W5).The second cluster contained the remaining two *C. colocynthis* acessions (W1 and W4) and all *C. lanatus* var*. lanatus* accessions (Fig. 6). The accessions collected from the same continent were not completely clustered in the same subclade indicating watermelon have occured different migrations and exchange between continents. Furthermore, we used a model-based approach for population structure analysis to analyze the germplasm panel of 134 accessions. According to distribution of ΔK values, there was only one peak of ΔK when K = 2 (ΔK = 202.11, Additional file 1: Figure S3) suggesting these 134 accessions were grouping into two populations (Fig. 7), which was almost completely consistent with the dendrogram. For example, W1 and W4 were two *C. colocynthis* acessions, but they were grouped with these *C. lanatus* var*. lanatus* accessions both in the genetic diversity and structure analysis. There were also three exceptions (W5, W9 and W12) which were clustered in group I in the dendrogram but sharing large admixed ancestry with *C. lanatus* var*. lanatus* population.

## Discussion

### Frequency, distribution and characterization of microsatellites in the watermelon genome

Discovery and mining of genomic SSR loci using whole genome sequences has had successful applications in a lot of plant species such as cucumber [23], cotton [34, 35], foxtail millet [24] and *Brachypodium* [36]. In the current study, a total of 39,523 microsatellites were identified from the watermelon genome assembly with a density of 111 SSR/Mb. The number of microsatellites and their density identified in our study was lower than that in cucumber (552 SSR/Mb) and *Arabidopsis* (371 SSR/Mb) [23], maize (120 SSR/Mb) and wheat (163 SSR/Mb) [37]. One main reason for these differences may be due to variations in the search parameters used for detection of microsatellites. For example, different repeat types (mono- to pentanucleotides versus mono- to octanucleotides) of different minimum lengths (12 bp versus 18 bp) were searched using different software. In this study, we analyzed the distribution and frequency of microsatellites with motifs of 2–8 bp long and minimum lengths of 18 bp or minimum of 3 repeat units in watermelon genome assembly. The criterion we used was based on the fact that polymorphism levels and mutation rate correlate positively with the number of repeat units [38], and therefore a higher polymorphic ratio is expected for these SSR markers developed in this study.

Frequency analysis of various nucleotide repeats in watermelon revealed that dinucleotide repeats were the most abundant SSRs followed by tri-, tetra-, penta-, hepta-, hexa- and octonucleotide repeats (Fig. 1 and Table 1). This was different with the trend in other species. For example, the tetranucleotide repeats were the most abundant in cucumber, *Medicago truncatula*, *Populus trichocarpa* and *Vitis vinifera*, and the trinucleotide repeats were the most abundant in *Glycine max*, *Arabidopsis thaliana*, *Oryza sativa* and *Sorghum bicolor* [23]. Overall, the AT-rich motifs such as AT and AAT were the predominant SSRs repeats types in each class in watermelon, representing 73.53 % and 74.55 % in dinucleotide repeats and trinucleotide repeats, respectively. Conversely, GC-rich repeat SSR motifs were very rare in all the nucleotides repeats. This result is consistent with other studies indicating that genomic SSRs with GC-rich repeats are rare in dicot species [39, 40].

The frequency and distribution of different SSR type in different chromosomes revealed that the frequency of microsatellite loci was not positively correlated with the chromosome size in watermelon. While 3486 SSRs were detected from the 27.02 Mb chromosome 6, only 2456 microsatellite loci were detected on chromosome 4 which has a similar size of 24.32 Mb (Additional file 2: Table S2). One important explanation was that there were highly enriched repeat sequences on the short arm of chromosome 4 [7]. This could be further confirmed in the 24.26 Mb unanchored scaffolds (chr0) on which only 889 microsatellite loci were detected. The remaining unassembled scaffolds from chr0 represented most of the repetitive fraction in watermelon genome. Our data is consistent with the observation in many plant species that SSR frequency is inversely to the proportion of repetitive DNA [41].

### In silico PCR analysis of cross-species transferability of SSRs in watermelon

Since SSR markers possess significant specificity and show a high percentage of cross-species transferability, they have been used for various genotyping applications including studying cross-transferability and comparative genome mapping in related species [42–44]. In the present study, we tested the genome wide of cross-species transferability of watermelon SSR markers in cucumber and melon by in silico PCR analysis. Among 32,869 SSRs examined, 832 (2.53 %) and 925 (2.81 %) had one non-redundant PCR product in cucumber and melon genome assembly, respectively. Although cucumber and melon have different chromosome numbers, they both belong to Cucumis genus sharing a common ancestor [33]. In our study, we found that the number of cross-species transferable SSR markers of watermelon was very close in cucumber and melon, which further confirmed their evolutionary relationship that cucumber and melon were closely related, but distantly related with watermelon. It should be pointed out that the frequency of cross-species SSR markers in cucumber and melon was probably underestimated for two reasons. First, the incomplete genome assembly of cucumber and melon which represent only 53 and 83 % of estimated cucumber and melon genome, respectively [27, 30]. Second, 59 and 44 SSR markers had multiply PCR products in cucumber and melon which were excluded for cross-species analysis. These cross-species transferable SSR markers covered 98.89 and 98.29 % of the cucumber and melon genome assembly, respectively. It should be noted that these cross-species transferable SSR markers had high density in both ends of watermelon chromosomes and only few of them were located in centromere regions (Fig. 3) which usually contains high repetitive sequence varying from species [43, 45]. These cross-species transferable SSR markers that generate one in silico PCR product should be the putative single-locus markers and could be especially useful in genetic map construction and gene mapping.

Furthermore, we investigated the frequency of different SSR motifs in 448 cross-three-species transferable SSR markers which were supposedly highly conserved. The frequency and distribution of microsatellite in these conserved 448 cross-species SSR markers were not consistent with that in the watermelon genome. For example, AG/CT was the most abundant motif in dinucleotides with a frequency of 59.12 % in the 448 cross-species SSR markers, while AT/TA (73.53 %) was the predominant motif in the dinucleotides in the watermelon genome and AG/CT was only accounting for 22.10 % of all dinucleotides (Additional file 1: Figure S1). In cucumber, AG/CT was the predominant dinucleotide in the EST-SSR [23] suggesting AG/CT was abundant in gene coding regions. This was confirmed by BLAST searching of cross-species transferable SSR markers in the CDS database of watermelon, and almost half of them were located in the coding regions. This might be because AG-rich regions are relatively stable, resulting in less replication slippage and usually distributed in exons, where polymorphisms occur less frequently [41]. Only 21 and 19 SSR markers were identified in watermelon EST and unigenes, respectively, which was probably due to the low coverage of EST collected in this database.

### Complicated syntenic pattern between watermelon withcucumber andmelon

Syntenic relationship revealed by comparative mapping has been carried out in a number of economically important plant families including the Poaceae, Solanaceae, Brassicaceae and Rosaceae [44, 46–48]. The studies on chromosome relationship in the Cucurbitaceae family largely focused on the *Cucumis* genus to understand the mechanisms of dysploid chromosome reduction from *n* = 12 to *n* = 7 [12, 27, 29, 31]. In the present study, the large number of cross-species SSR markers provided a good opportunity to uncover the syntenic relationships among watermelon, melon and cucumber genomes at a high resolution level which enabled us to detect different patterns of chromosome evolution for three cucurbit species. We identified 84 and 81 watermelon-cucumber and watermelon-melon syntenic blocks, respectively. In most cases, each watermelon chromosome showed major synteny to at least three cucumber chromosomes and three melon chromosome, respectively (Table 3 and Fig. 3), indicating that these chromosomes have undergone chromosome fission after its divergence from the ancestor leading to cucumber and melon. Watermelon chromosome W3 and W10 had the simplest syntenic pattern to cucumber with each of them only corresponding to two cucumber chromosomes. For example, 86 of shared SSR markers on watermelon W10 were mapped on cucumber C3, and five SSR markers were syntenic to cucumber C4. In-depth analysis revealed the shared SSR markers were further divided into three collinear blocks (WCB68, 70 and 72) and three inverted blocks (WCB67, 69 and 73) suggesting the very complicated evolutionary dynamics of chromosomes evolution in the Cucurbitaceae family. Although watermelon (*n* = 11) and melon (*n* = 12) have similar chromosome number, the syntenic patterns were not simple one to one chromosome. Most watermelon chromosome were syntenic to three or four melon chromosomes, indicating the chromosome rearrangements between melon and watermelon were much more complicated compared with other closely related species with similar chromosome number in the same family. For example, in Rosaceae, most of peach (*n* = 8) chromosome showed major synteny to one strawberry (*n* = 7) chromosome [46]. This implies that much more structural changes between melon and watermelon have occurred during their karyotype evolution, speciation and local adaptation.

The evolutionary relationships among three cucurbit species were further analysed by investigating the syntenic blocks in watermelon, melon and cucumber. 25 syntenic blocks were identified that were shared by the three genomes (Additional file 2: Table S6), which covered about 57 Mb representing 17.08 % of watermelon genome assembly, suggesting these genomic regions may be highly conserved during chromosome evolution in the Cucurbitaceae family. Compared with these conserved blocks, other syntenic blocks were split into small blocks or rearranged into new blocks between three genomes. For example, the WCB6 on watermelon chromosome W1 (26.13–33.99 Mb) was collinear to cucumber chromosome C6, which was divided into two block in melon (WMB5 and WMB6) (Additional file 2: Table S6). This phenomenon was further confirmed by comparative pachytene FISH on chromosome synteny of cucumber C7, melon I and watermelon W2 and W9. Previous studies have revealed that cucumber C7 was highly conserved with melon I and even in other farther related species of *Cucumis* genus, and mainly syntenic to watermelon chromosomes W2 and W9 [7, 12]. The large syntenic block WCB62 was an inversion between cucumber C7 and watermelon W9 confirmed by fosmid probes 71.7–72.4, while it was splite to several small inverted blocks in melon genome (Fig. 5 and Additional file 2: Table S3), indicating this genomic region have undergone extra structure changes in melon lineage after its divergence from the common ancestor of *Cucumis*. In addition, the syntenic blocks identified in this study will also help to improve the genome assembly. For example, only one markers ClSSR03519 in block WMB6 developed from scaffolds unanchored to chromosomes in melon, while 26 shared SSR markers in this block were highly continuously distributed on watermelon chromosome W1 and melon chromosome VIII, suggesting the scaffold from which ClSSR03519 was developed should be anchored on melon chromosome VIII.

### The genetic diversity and population structure of watermelon

Due to the scarcity of highly polymorphic, user-friendly molecular markers in watermelon, high-density genetic maps were not available until recently [8]. Most genetic diversity and linkage maps have been used low throughput, anonymous, dominant markers such as RAPDs, AFLPs and SRAPs in watermelon [49, 50], making them difficult in applications like map-based cloning, and maker-assisted selection. The large number of genome wide SSR markers developed from watermelon genome in this study should provide a valuable resource to the watermelon community in various marker-based studies such as genetic diversity analysis, gene mapping or cloning, genome wide association study (GWAS) and marker-assisted selection for watermelon breeding. By validating 192 SSR markers (Additional file 2: Table S8), as high as 47.40 % of them were polymorphic in six accessions of *C. lanatus* var*. lanatus,* suggesting these SSR markers will be useful in genetic diversity study. Furthermore, the cross-species transferable SSR markers identified by in silico PCR analysis in cucumber and melon genome could also be applied in studies of development of practical markers in other closely related species of same genus.

In our study, 32 highly informative SSR markers were used for genetic diversity anlaysis and inferring population structure of 134 watermelon accessions revealed that they were divided into two groups in the UPGMA tree which were consistent with their population structure (Fig. 6 and Fig. 7). Interestingly, two *C. colocynthis* accessions W1 and W4 were clustered with all *C. lanatus* var*. lanatus* accessions in group II, and population structure also revealed that they shared a large part background with *C. lanatus* var*. lanatus,* suggesting this two *C. colocynthis* accessions are more closely related to *C. lanatus* var*. lanatus* (Fig. 7)*,* which is especially true for the accession W1 (PI 388770). PI 388770 was also used previously by Levi et al. [6], who also found that this accession was closer to *C. lanatus* var*. lanatus*. Therefore these two accessions may merit additional investigations to resolve the anomaly. The structure analysis revealed that K = 2 was the best value for classfication of the 134 watermelon accessions which was different with other studies. Levi et al. [6] used high frequency oligonucleotides targeting active gene (HFO-TAG) markers grouping 96 watermelon accessions into four population, while Reddy et al. [9] grouped 96 different watermelon accessions into five population revealed by 201 SSR markers. This suggested the population structure of watermelon varied by using different accessions and different molecular marker numbers.

Watermelon has a very narrow genetic base and evaluating the phylogenetic relationships among different species in *Citrullus* genus will help us for better improving the watermelon cultivars and broaden its gene pool specially from the primary gene pools: *C. lanatus* var. *lanatus*, *C. lanatus* var*. citorides* and *Citrullus colocynthis* [2, 5, 51]*.* For example, three accessions (W8, W9 and W15) of *C. lanatus* var*. citorides* were previously reported resistant to anthracnose race 2 [52], one accession W11 was reported to contain resistance to Fusarium wilt [53] and another accession W1 of *C. colocynthis* showed fruit rot resistance [54]. These sources of resistance to different diseases will be valuable in watermelon breeding programs aimed at enhancing disease resistance. In addition, the large differentiation among populations indicates that each population may possess its own alleles and haplotypes. Therefore, crosses between different populations will broaden the genetic diversity within current breeding programs and may increase heterosis.

## Conclusions

We developed genome wide microsatellite and characterized the frequency and distribution of different motifs in watermelon. The dinucleotides were the most abundant type and AT-rich motifs were predominant motifs in all nucleotide repeat in watermelon genome. Furthermore, the cross-species transferable SSR were detected in melon and cucumber by in silico PCR analysis, and these large number share SSR markers provide a higher level of resolution for comparative mapping to understand genomic relationships among these three species in the Cucurbitaceae family. Most of the chromosome in melon and cucumber were syntenic to three or four chromosomes of watermelon. The chromosome synteny suggested complicated structure rearrangements occurred from watermelon to melon and cucumber after their divergence from common ancestor. In addition, 32 high polymorphism SSR markers were used to study the genetic diversity of 134 watermelon accessions which were clustered into two groups. The large number SSR markers in watermelon and cross-species transferable SSR identified in this study could be applied in many research areas such as map construction, comparative mapping and marker-assisted trait selection, and also provide an important marker resource to other closely related and genome unsequenced species in Cucurbitaceae family.

## Methods

### Plant material and DNA isolation

A total of 134 watermelon Plant Introduction (PI) accessions from diverse geographic regions were selected for genetic diversity analysis (Additional file 2: Table S1). Of them, 117, 12 and 5 are designated as *C. lanatus* var. *lanatus*, *C. lanatus* var. *citroides* and *C. colocynthis*, respectively. Geographically, 62 were from Asian, 4 from Europe, 40 from Africa, 25 from North America and 3 from South America. Unexpanded young leaves from these accessions were collected into 2.0 mL microcentrifuge tubes, lyophilized in a freeze dryer, and ground into fine powder. Genomic DNA was extracted using the CTAB method [55].

### SSR identification and primer design

The genome assembly of watermelon were downloaded from Cucurbit Genomics Database (http://www.icugi.org/cgi-bin/ICuGI/index.cgi). To develop a higher polymorphism SSR primer set for future study, the criteria used for microsatellite identification in this study was from 2- to 8-bp motifs, and mononucleotides were not considered due to the difficulty of distinguishing bona fide microsatellites from sequencing or assembly error. DNA sequences were searched for both perfect and compound microsatellites, with a basic motif of 2–8 bp, using the computer program MISA (Microsatellite identification tool) [56]. Repeats with a minimum length of 18 (for di- to tetranucleotides), 20 (for pentanucleotides), 24 (for hexanucleotides), 21 (for heptanucleotides), and 24 bp (for octanucleotides) were recorded. The physical positions of the SSRs found in the chromosomes were also recorded, and oligonucleotide primers were designed for the genomic sequence flanking these SSRs using Primer3 (v. 1.1.4) software [57]. Primers were designed to generate amplicons of 100–300 bp in length with the following minimum, optimum and maximum values for Primer3 parameters: primer length (bp): 18-20-24; Tm (°C): 50-55-60. Other parameters used the default program values.

### In silico analysis of watermelon SSR markers in cucumber and melon genome

Using an in silico PCR strategy, all the SSR primer developed from watermelon genome assembly were used to BLAST search in cucumber Gy14 [27] and melon DHL92 genome assembly [30] which were downloaded from http://wenglab.horticulture.wisc.edu/cucumber-genome-database/ and https://melonomics.net, respectively. This was performed with a custom Perl script that used the NCBI BLASTN program as a search engine with expect value of 10 and filtering. We allowed up to 5 nucleotide mismatches at the 5′ end of the primer but no mismatches at the 3′ end, and a minimum of 90 % overall match homology. The in silico PCR products containing single copy or multiple copies were both recorded for further analysis. Furthermore, the set of cross-three-species transferable SSR were selected by comparing common SSR markers between watermelon with melon and cucumber. To investigate the distribution of these highly conserved SSR markers in watermelon, the in silico PCR product sequences were used to blast search in EST, unigene and CDS database of watermelon from the cucurbit genomics database (http://www.icugi.org) with a threshold E-10.

To establish the syntenic chromosomes relationships between watermelon with cucumber and melon, we only kept the SSR markers in melon and cucumber genomes which had single in silico PCR product. In addition, these shared SSR markers located on the chromosomal unanchored scaffolds were further filtered. Then the chromosome relationship among three species was inferred by the remaining shared SSR markers. The SSR marker-based syntenic relationships among cucumber, melon and watermelon were finally visualized with visualization blocks in Circos software v 0.55 (http://circos.ca) [58].

### PCR amplification and validation of selected SSRs

One hundred and ninety-two SSR markers were selected to validate the polymorphism in six accessions of watermelon, and 32 of them with informative and unambiguous bands were further chosen in the genetic diversity study. Each polymerase chain reaction (PCR) contained 25 ng template DNA, 0.5 μM each of forward and reverse primers, 0.2 mM dNTP mix, 0.5 unit of Taq DNA polymerase and 1× PCR buffer in a total volume of 10.0 μl. The amplification was carried out at initial denaturing step at 94 °C for 4 min followed by 30 cycles of 94 °C for 20 sec, 55 °C for 45 s and 72 °C for 1 min. In the last cycle, primer extension was performed at 72 °C for 10 min and storage at 4 °C till electrophoresis. The PCR products were size-fractionated in a 9 % polyacrylamide gel. The 100-bp DNA ladder was used as molecular size marker. After gel electrophoresis, band patterns were visualized with silver staining, and gel images were taken with a digital camera.

### Genetic diversity and population structure analysis

The genomic DNA fragments from SSRs generated clear and unambiguous bands of various molecular weight sizes were scored for watermelon 134 accessions and calculated into co-dominant genotypic matrix in GeneAlEx 6.5 [59], then the UPGMA method was used to construct the dendrogram by software MEGA5 [60]. The observed (Na) and effective (Ne) number of alleles, Shannon’s information index (I), and levels of observed (Ho) and expected (He) heterozygosity were calculated by GeneAlEx 6.5. Polymorphic information content (PIC) for molecular markers was calculated as par the formula: PIC = ΣPij^2^ where Pij is the frequency of the j^th^ pattern for marker j and the summation extends over n patterns.

The model-based program STRUCTURE was used to infer population structure by the program STRUCTURE 2.3 [61, 62]. The program was run with SSR markers for k-values from 1 to 10, and the number of populations (K) was determined using an admixture model with correlated alleles. Twenty independent runs of 100,000 Markov Chain Monte Carlo generations after 50,000 generation burn-in periods were used to estimate each value of K. The optimal K depends on the peak of ΔK = mean (|Ln"P(D)|)/(sdLnP(D)), where|Ln"P(D)|denotes the absolute value of the second order rate of change of LnP(D) and sdLnP(D) the standard deviation of the LnP(D). To infer the true K, we run another twenty independent runs for the K from 1–5 with 750,000 Markov Chain Monte Carlo generations after 500,000 generation burn-in periods.

### Comparative fluorescence in situ hybridization (FISH)

To examine and validate chromosome rearrangements between cucumber C7, melon I, watermelon W2 and W9, 14 fosmid probes identified on cucumber C7 [12] were used in comparative FISH mapping of meiotic pachytene chromosomes prepared from pollen mother cells of melon and watermelon. The physical order of adjacent fosmid clones in each chromosome was determined by two-color FISH. The FISH procedure was performed as described by Koo et al. [63]. Biotin- and digoxigenin-labeled probes were detected with Alexa Fluor 488 streptavidin antibody (Invitrogen, Carlsbad, CA) and rhodamine-conjugated anti-digoxigenin antibody (Roche Diagnostics USA, Indianapolis, IN), respectively. Chromosomes were counterstained by 4¢, 6-diamidino-2-phenylindole (DAPI) in ‘Vector Shield’ antifade solution (Vector Laboratories, Burlingame, CA). FISH signals were captured using a CCD camera. The images were processed using Meta Imaging Series 7.5 software (Molecular Devices, Downingtown, PA, USA).

## Abbreviations

AFLP, amplified fragment length polymorphism; BLAST, basic local alignment search tool; CDS, coding sequence; EST, expressed sequence tag; FISH, fluorescence in situ hybridization; GWAS, genome wide association study; HFO-TAG, high frequency oligonucleotides targeting active gene; MAS, marker-assisted selection; Mb, million base pairs; MISA, microsatellite identification tool; NGS, next-generation sequencing; PCR, polymerase chain reaction; PI, plant introductions; PIC, polymorphic information content; QTL, quantitative trait loci; RAPD, random amplified polymorphic DNA; SNP, single nucleotide polymorphism; SRAP, sequence related amplified polymorphism; SSR, simple sequence repeats; WGS, whole-genome sequences

## Additional files

Additional file 1:
**Figure S1.** The motif distribution of dinucleotide (A), trinucleotide (B) and tetranucleotide (C) in watermelon. Figure S2. The chromosome position of the 32 SSR markers in watermelon. Figure S3. Delta K distribution across various clusters (K) as estimated by Structure Harvester. (PDF 833 kb) Additional file 2:
**Table S1.** Watermelon Plant introduction accessions (PIs) used in the present study. Table S2. Genome wide SSR primers developed in the watermelon genome. Table S3. List of cross-species SSR markers between watermelon and cucumber identified by in silico PCR. Table S4. List of cross-species SSR markers between watermelon and melon identified by in silico PCR. Table S5. List of conserved SSR markers among watermelon, melon and cucumber genomes. Table S6. Syntenic blocks shared for cucumber, melon and watermelon genomes. Table S7. Information of fosmid clones used in fluorescence in situ hybridization (FISH). Table S8. List of 192 SSR primers used for polymorphism screening in watermelon. Table S9. Statistic of observed (Na) and effective number of alleles (Ne), observed (Ho) and expected heterozygosity (He), polymorphic information content (PIC) and Shannon’s information index (I) for 32 SSR markers. (XLSX 3673 kb)
