# Supplementary figures and images for: Genome wide characterization of simple sequence repeats in watermelon genome and their application in comparative mapping and genetic diversity analysis
Source: BMC Genomics. 2016 Aug 5;17:557. doi: 10.1186/s12864-016-2870-4 (PMC4974753; doi:10.1186/s12864-016-2870-4)

Figure S1

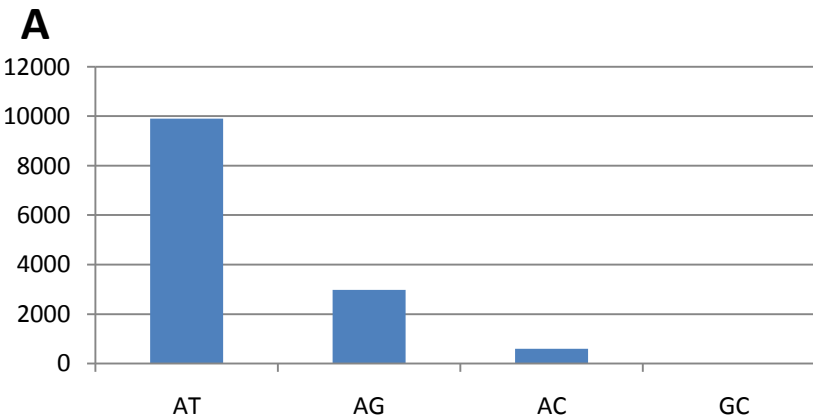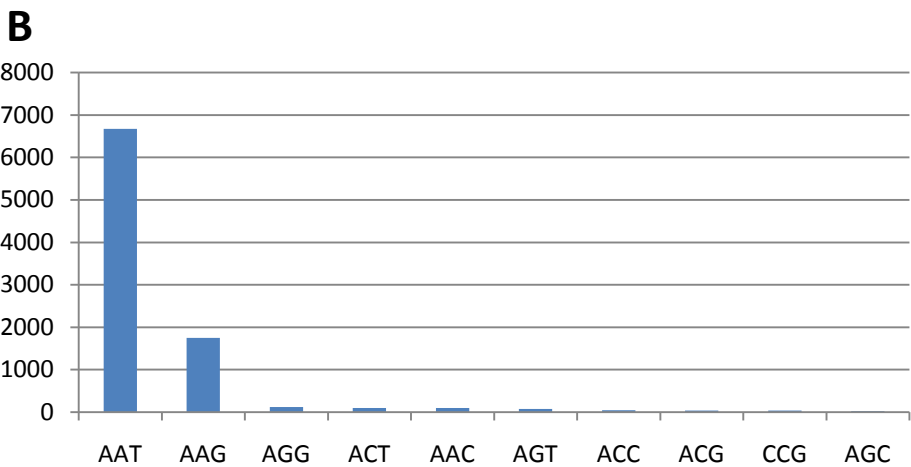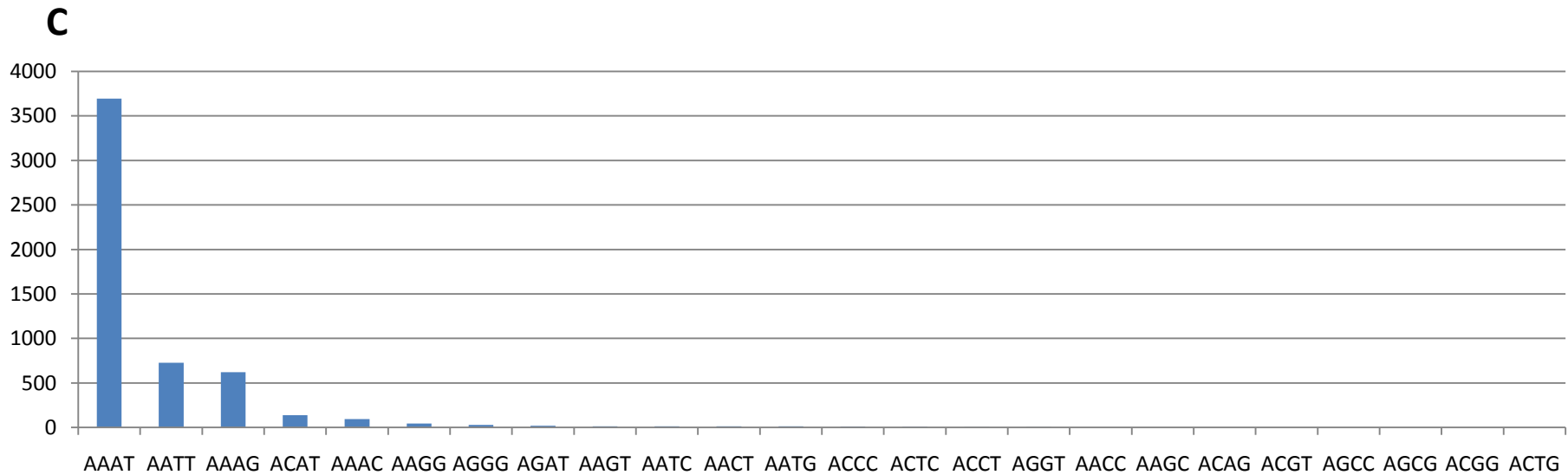

Figure S2

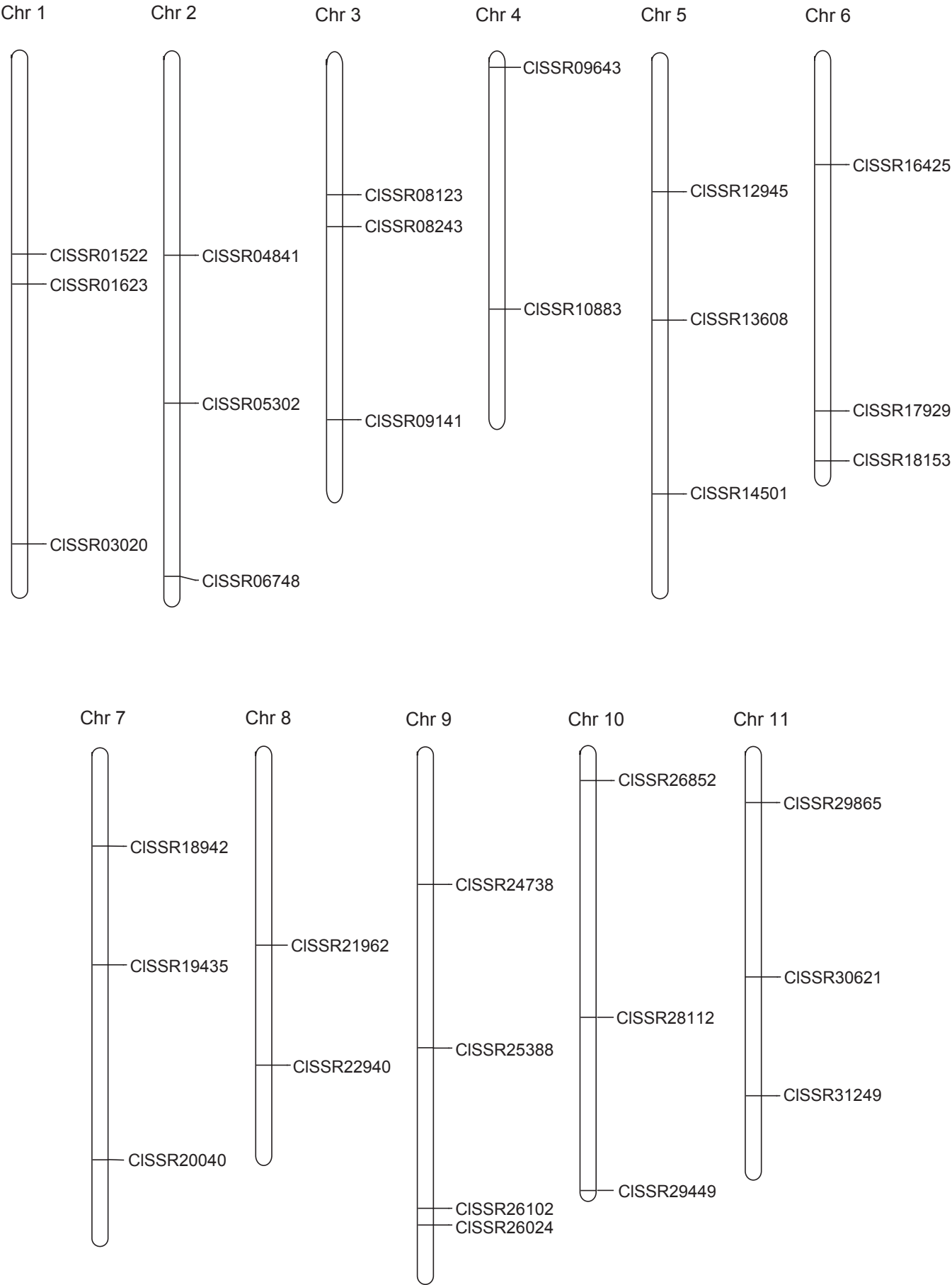

**Figure S3**

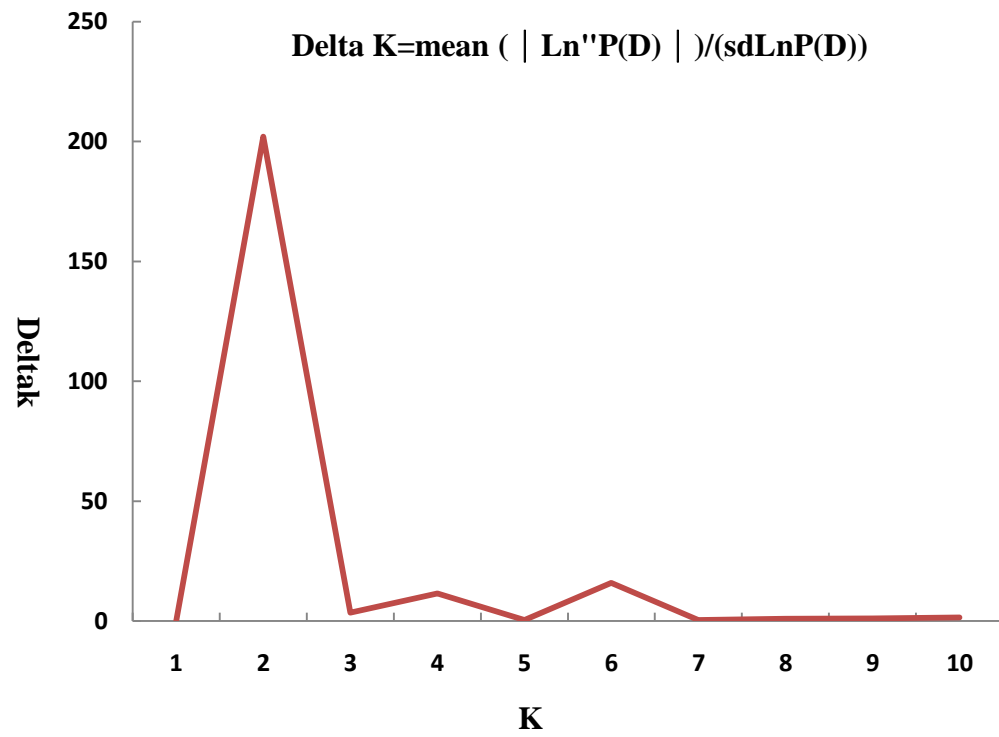

Supplement: Additional file 1: — Figure S1. The motif distribution of dinucleotide (A), trinucleotide (B) and tetranucleotide (C) in watermelon. Figure S2. The chromosome position of the 32 SSR markers in watermelon. Figure S3. Delta K distribution across various clusters (K) as estimated by Structure Harvester. (PDF 833 kb) [file 12864_2016_2870_MOESM1_ESM.pdf]
